# Association of 12 serum biochemical markers of angiogenesis, tumour invasion and bone turnover with bone metastases from breast cancer: a crossectional and longitudinal evaluation

**DOI:** 10.1038/sj.bjc.6603285

**Published:** 2006-08-01

**Authors:** N Voorzanger-Rousselot, F Juillet, E Mareau, J Zimmermann, T Kalebic, P Garnero

**Affiliations:** 1 Molecular Markers, Synarc, 16 rue Montbrillant Le Buroparc T4, 69416, Lyon, Cedex 03, France; 2 Novartis Oncology, Florham Park, NJ 07932, USA; 3 Faculte de Medicine-RTH Laënnec, INSERM Research Unité 664, Lyon 69372, France

**Keywords:** breast carcinoma, bone metastases, bone markers, matrix metalloproteinases, prognosis

## Abstract

Complex biological pathways including angiogenesis, invasion, osteoclastic activation and bone matrix degradation are involved in the formation of bone metastasis (BM). The aim of our study was to investigate the cross-sectional and longitudinal associations of a panel of 12 serum biochemical markers reflecting biological pathways underlying BM development. In a cross-sectional study, we investigated 29 patients with primary breast carcinoma without BM (BC/BM−), 28 patients with breast carcinoma and BM (BC/BM+) and 15 healthy women. In longitudinal analyses, we investigated 34 patients for whom serum was obtained a two different time points: at the time of primary BC diagnosis and after a median time of 3 years. During this follow-up, 15 patients developed BM, whereas the other 19 remained free of BM. In patients who developed BM, the second samples were obtained before BM was documented by bone scan. The cross-sectional analyses have shown all biochemical markers to be significantly elevated in patients with BM, when compared to the patients without BM and healthy controls, except TGF*β*1 that was significantly decreased. Multivariable analyses showed that only the bone resorption markers TRACP 5b, CTX and ICTP, and the marker of angiogenesis VEGF were independently associated with BM. Those markers correctly distinguished 85% of BC patients with or without BM from normal individuals. Longitudinal analyses showed that patients with primary BC who developed BM during follow-up had higher levels of TRACP5b (+95%, *P*=0.08) at the time of primary diagnosis, those patients had also a higher increases of ICTP (*P*=0.006), MMP-7 (*P*=0.004) and TIMP-1 (*P*=0.017) during follow-up than patients who did not progress toward bone metastasis. This study provides evidence of increase and interrelationship of circulating markers of angiogenesis, invasion and bone resorption in patients with BC with and without BM. Markers of bone resorption have the highest independent diagnostic value for detecting and potentially predicting BM in breast carcinoma patients.

Bone is one of the most common sites of metastasis for certain types of tumours, especially prostate, breast and lung carcinoma (Mundy, 2002). Breast cancer progression to bone is a defining feature of a highly malignant tumour and the major cause of cancer-treatment failure. Early detection of bone metastasis is critical for clinical management and accurate staging of tumours. Currently there is no a simple way to reliably detect and predict which patients will develop bone metastasis. The detection of bone metastases relies on imaging technology, but novel approaches based on biomarker assessments are under intense investigation. Increasing body of data suggests that metastatic dissemination is site specific (Horak and Steeg, 2005) and that distinct molecular factors regulate bone metastasis formation (Yin *et al*, 2005). A multifactorial and multistep process of bone metastasis formation involves several biological mechanisms including angiogenesis, invasion through extracellular matrix degradation, osteoblast/osteoclast activation and bone remodeling activity (Guise and Mundy 1998). Angiogenesis or blood vessel formation occurs in both physiological and pathological conditions, such as metastatic formation (Liotta *et al*, 1991; Folkman, 2003). Molecular factors regulating angiogenesis have been found also implicated in the regulation of tumour invasion (Kalebic *et al*, 1983). The number and density of microvessels in different human cancers was found associated with their invasive and metastatic potential (Kato *et al*, 2003) and have been shown to correlate with the serum concentrations of angiogenic factors such as the vascular endothelial growth factor (VEGF) (Coskun *et al*, 2003). High circulating levels of VEGF have been found to correlate with more advanced stages or with a worse prognosis in different tumours and in particular in women with breast cancer (Linderholm *et al*, 2000). Histological studies of bone metastases show that tumour cells remain in the bone marrow cavity and secrete factors that regulate bone cells including parathyroid hormone related protein (PTHrP), tumour necrosis factor (TNF), interleukin-6 (IL-6) and transforming growth factor (TGF)*β* (Yin *et al*, 2005). Among these local factors, serum TGF*β* was recently shown to be associated with disease progression and poor prognosis in patients with metastatic breast cancer (Cui *et al*, 1996; Sheen-Chen *et al* 2001; Ivanovic *et al*, 2003). Matrix-metalloproteases (MMPs) are a family of zinc-dependent endopeptidases, which degrade the extracellular matrix proteins. Increased levels of MMPs have been found associated with basement membrane invasion, which plays an important role in metastasis formation (Kalebic *et al*, 1983; Stetler-Stevenson, 1994, 1995; Kleiner and Stetler-Stevenson, 1999). The activity of MMPs is regulated by tissue inhibitors (TIMPs) (Chenard *et al*, 1996; Duffy *et al*, 2000; Hirvonen *et al*, 2003). Among the different MMPs, it has been shown that increased expression of MMP-2 was associated with early relapse and short survival (Talvensaari-Mattila *et al*, 1998; Talvensaari-Mattila *et al*, 1999). The coexpression of MMP-2 and MMP-9 provided unfavourable prognostic value in node-negative breast cancer patients (Li *et al*, 2004).

Bone metastases from breast cancer induce alterations of bone remodelling activity mediated by cytokines and growth factors secreted in the bone microenvironment by cancer cells. Increased osteoclastic bone resorption is likely to play a major role in mainly lytic features of bone metastases process. The pivotal regulator of osteoclastic activity is the system osteoprotegerin (OPG)/receptor activator of NF-*κ*B (RANK)/RANK-ligand (RANKL) (Hofbauer *et al*, 2004), which is involved in physiological as well as pathological conditions, such as metastatic bone disease and multiple myeloma (Chikatsu *et al*, 2000; Lipton *et al*, 2002; Park *et al*, 2003; Vanderkerken *et al*, 2003). Increase levels of OPG but not RANKL have also been reported in patients with prostate cancer and bone metastases (Jung *et al*, 2004).

The markers of bone turnover, which include enzymes predominantly expressed by the osteoblasts, such as bone alkaline phosphatase (bone ALP), and osteoclasts – such as the tartrate resistant acid phosphatase, (TRACP) – or bone matrix synthesis and degradation products (Fontana *et al*, 1999) have demonstrated increased serum and urine levels in patients with bone metastases (Demers *et al*, 2000). Our previous study has shown that bone matrix degradation markers especially the type I collagen C-telopeptides fragments CTX and ICTP, which reflect cathepsin K- and MMP-mediated type I collagen degradation, respectively, could be useful in monitoring bone metastasis (Garnero *et al*, 2003a).

Most of the previous studies evaluated only one or a few of the above described biochemical markers in cancer patients. Those markers were usually linked to a particular biological process and the sensitivity to detect bone metastases was rather limited. The aim of our study was to measure a panel of systemic biochemical markers associated with multiple biological processes involved in the formation of bone metastases. We have assessed those markers in patients with the primary breast carcinoma with or without bone metastases and investigated whether a combination of markers could be used as a novel tool to detect and predict bone metastasis in breast carcinoma patients.

## MATERIALS AND METHODS

### Patients

Fasting morning serum samples from patients with breast carcinoma (BC) and healthy age-matched women were provided by ASTERANG (Detroit, USA) and Clinomics Biosciences (New York, NY, USA), respectively. All serum samples were kept frozen at −70°C until conducting the assay. All subjects gave written informed consent to participate in the study, which was carried out in accordance with the Helsinki declaration. The main characteristics of patients with and without bone metastases with respect to tumour size, stage, tumour histology, nodal status, oestrogen receptor (ER) status and therapy are shown on Table 1.

The following groups of patients were investigated in cross-sectional and longitudinal analyses

#### Crossectional study

- Twenty-nine postmenopausal women with primary breast cancer without bone metastases were included. The majority of patients presented with infiltrating ductal carcinoma. Bone survey with Tc-99 bone scan and X-ray showed no bone metastases. Patients were also free of nonbone metastases.
- Twenty-eight postmenopausal women with primary breast cancer and radiologically confirmed bone metastases. The majority of patients presented with infiltrating ductal carcinoma. Among these patients 4 (14%) had also lung metastases.
- Fifteen healthy postmenopausal women (mean age: 59±6 years) with no history of breast disease or metabolic bone disease.

#### Longitudinal study

- Thirty-four postmenopausal women with breast cancer without bone metastases at the time of diagnosis were followed for a median of 3 years (1–6 years). Bone scintigraphy was performed every 3–6 months in all patients to monitor appearance of bone metastases. Among these 34 patients, 19 patients remained free of bone whereas the other 15 developed bone metastases.
- In the 19 patients who remained free of bone metastases, the second samples were obtained after 3 years in 14 of the patients and after 6 years in the others. During this period, none of these patients developed nonbone metastases. At the time of first sampling, four (21%) received chemotherapy, three (16%) had radiotherapy and information on treatment was not available in the rest.
- For patients who developed bone metastases, the second sample was obtained before bone metastases were documented by bone scans. The delay between the second measurement and diagnosis of bone metastases on bone scan was 1 month for nine patients, 2 months for two patients, 4 months for two patients and 6 months for two patients.
- None of these patients developed nonbone metastases. At the time of first sampling, four (27%) received chemotherapy, four (27%) hormonal treatments and information on treatment was not available in the rest.

### Biochemical markers

#### Growth factors

Serum VEGF was measured by an ELISA recognising both VEGF165 and VEGF121 isoforms (Quantikine® R&D, Minneapolis, USA). The intra and inter assay precision errors are 7 and 8%, respectively. Serum TGF*β*1 was measured by a two site ELISA using specific antibodies raised against human recombinant TGF*β*1 (IBL, Hamburg Germany). The intra and inter assay precision error are below 1.4 and 11%, respectively.

#### Osteoclastogenesis markers

Serum OPG was measured by a two site immunoassay using antibodies raised against recombinant human OPG (Biomedica, Vienna, Austria). Intra and inter assay variations are lower than 10 and 13%, respectively. Serum RANK-L was measured by an immunoassay based on a sandwich between coated OPG and a polyclonal antibody raised against recombinant human RANK-L (Biomedica, Vienna, Austria). The intra- and inter-assay variation are lower than 10%.

#### Matrix-metalloproteases

Serum MMP 2, 7 and 9 were measured by two site ELISAs (Quantikine® R&D, Minneapolis, USA). The intra- and inter-assay precision errors are 2 and 8 % for MMP-2, 2.2 and 6%, for MMP-7 and 7 and 11%, for MMP-9. Serum TIMP-1 was measured by a two site ELISA using specific antibodies raised against human recombinant TIMP-1 (Quantikine® R&D, Minneapolis, USA). The intra and inter assay precision errors are below 3 and 10%, respectively.

### Bone turnover markers

#### Bone formation markers

Serum bone ALP was measured by an immunochimiluminescence assay using the Ostase reagent on an automatic analyzer (Ostase, Access, Beckman Coulter, Fullerton, CA, USA). The intra and inter assay CV are below 3.5 and 8%, respectively. The cross-reactivity of the assay with the liver isoenzyme is of 13%. Serum intact procollagen type I N propeptide (PINP) was measured with a two site immunoassay based on monoclonal antibodies raised against purified intact human PINP and detecting both intact mono and trimeric forms, but not fragments using an automated analyzer (Elecsys, Roche Diagnostics, Mannheim, Germany). Intra-assay variation is lower than 3% and inter-assay variation lower than 4%.

#### Bone resorption markers

Serum C terminal crosslinking telopeptide of type I collagen (S-CTX) was measured by a two site assay using monoclonal antibodies raised against an eight amino-acid sequence from the C-telopeptide of human type I collagen by an automatic analyzer (Elecsys, Roche Diagnostic, Mannheim, Germany). Intra-assay variation is lower than 3% and inter-assay variation is lower than 5%. Serum C-terminal crosslinking telopeptide of type I collagen generated by MMPs (ICTP) was measured by a radioimmunoassay (Telopeptide ICTP, Orion Diagnostica, Espoo, Finland). The intra- and inter-assay CVS are below 5 and 7%, respectively. Serum TRACP isoform 5b (TRACP 5b) was measured by a specific immunoassay using a monoclonal antibody raised against TRACP 5b purified from human osteoclasts and recombinant human TRACP5b as a standard (SBA Sciences, Turku, Finland). Intra- and inter-assay coefficients of variation are lower than 10%.

### Statistical analysis

Statistical analyses were performed using the Statistical Analysis System 8e (SAS Institute Inc., Cary, NC, USA). Differences of biochemical marker levels between patient groups were analysed using the nonparametric Kruskal–Wallis test, followed by Mann–Whitney or Wilcoxon tests for two-group comparisons. Relationships between biochemical markers were assessed using the Spearman (rank) correlation analysis. Diagnostic accuracy was evaluated by receiving operating curve (ROC) analysis. Sensitivity and specificity to detect bone metastases were calculated using the cutoff level with the highest diagnostic accuracy obtained from ROC analysis. Diagnostic accuracy was calculated as the proportion of patients with (true positive) or without (true negative) bone metastases correctly classified.

A discriminant analysis was performed to determine the optimal combination of biochemical markers to distinguish patients with and without bone metastases. In this model, biomarkers were used as quantitative continuous variables and the two groups (with or without metastases) as a dichotomous classification variable. A stepwise discriminant analysis (stepwise backward elimination) was performed to select a subset of the quantitative continuous variables for use in classifying patients between the two groups. The significance level allowing to the markers not to be excluded from the model was 0.15.

Under the hypothesis of a multivariate normal distribution within each group, the discriminant function was determined by a measure of generalised squared distance. A performance of the discriminant criterion was evaluated by estimating error rates in the classification of future observations.

In longitudinal analysis, for each patient the changes during follow-up in biochemical marker levels were calculated as ((follow-up value−baseline value)/baseline value) adjusted for the time of follow-up. Baseline and changes in biochemical markers between patients who developed and those who did not develop bone metastases during follow were compared using nonparametric Mann–Whitney test.

## RESULTS

### Levels of biochemical markers of angiogenesis, osteoclastogenesis and bone turnover in breast carcinoma patients with and without bone metastases

**Figure 1:**
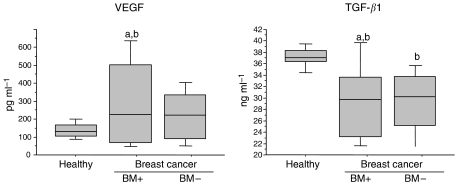
Box plots of serum VEGF and TGF*β*1 levels in patients with breast cancer with (BM+, *n*=28) or without (BM−, *n*=34) bone metastasis and healthy controls (*n*=15). From the bottom-up, the box indicates the 25th, 50th (median) and 75th percentiles, while the bars indicate the 10th and 90th percentiles, respectively. Significance levels were obtained from non parametric Mann–Whitney test: (a) *P*<0.05: *vs* BM- , (b) *P*<0.05: *vs* healthy controls.

**Figure 2:**
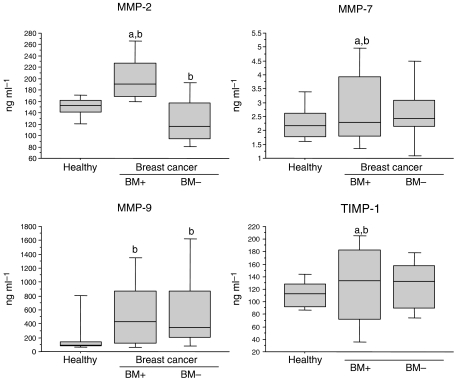
Box plots of serum matrix metalloproteinases MMP-2, MMP-7, MMP-9 and tissue inhibitor of MMPs (TIMP-1) levels in patients with breast cancer with (BM+, *n*=28) or without (BM−, *n*=34) bone metastasis and healthy controls. For details, see legend of Figure 1.

**Figure 3:**
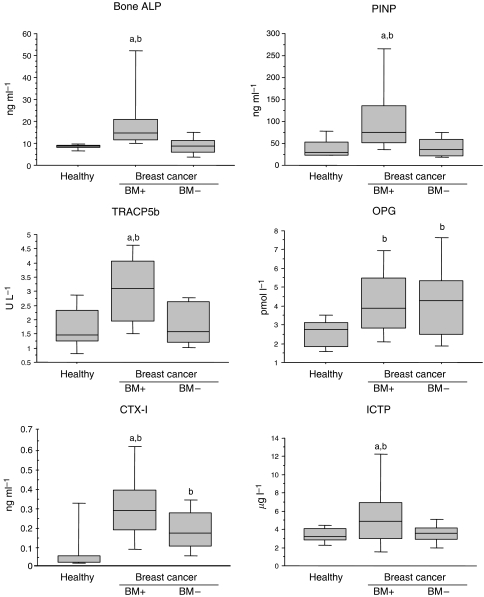
Box plot distribution of serum bone formation (bone ALP, PINP), bone resorption (TRACP5b, CTX-I, ICTP) and osteoclastogenesis (OPG) markers levels in patients with breast cancer with (BM+, *n*=28) or without (BM−, *n*=34) bone metastasis and healthy controls (*n*=15). For details, see legend of Figure 1.

All biochemical markers were significantly increased in patients with breast cancer with bone metastases, when compared to either the patients with breast cancer without bone metastases or healthy controls, except for TGF*β*1 levels, which were significantly decreased (*P*=0.05 and *P*=0.005, respectively) (Figures 1, 2 and 3).

In contrast, patients with breast cancer without bone metastases and healthy controls have shown a comparable level of biomarkers. However, slightly lower levels of TGF*β*1 (*P*<0.0001) and MMP-2 (*P*=0.03) and a small increase of MMP-9 (*P*=0.001), OPG (*P*=0.003) and CTX (*P*=0.0007) have been detected in breast carcinoma patients (Figures 1, 2 and 3).

No significant difference among three groups in serum RANK-L (data not shown) has been observed. These data need to be interpreted with caution because the number of undetectable values was high (13, 79 and 33% in healthy controls, patients with breast cancer and without bone metastases, patients with breast cancer and with bone metastases, respectively), and this marker was no considered in the subsequent statistical analyses.

### Relationships between biochemical markers

**Figure 4:**
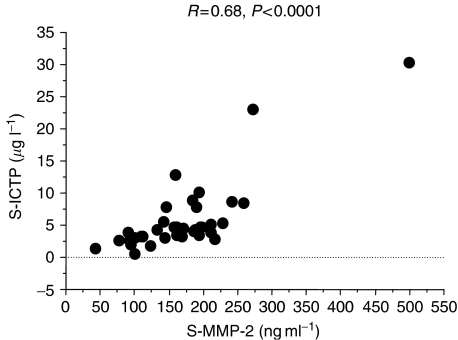
Correlation between serum MMP-2 and serum ICTP in patients with breast cancer with or without bone metastases included in the cross-sectional analysis.

In women with breast cancer, there were significant correlations between all bone turnover markers (*r* values ranging from 0.26 to 0.78, *P*<0.05–0.0001). Interestingly, among the two type I collagen degradation markers, ICTP showed higher associations with VEGF (*r*=0.37, *P*<0.01), MMP-2 (*r*=0.68, *P*<0.0001) (Figure 4), MMP-7 (*r*=0.53, *P*<0.0001), TIMP-1 (*r*=0.63, *P*<0.0001), Bone ALP (*r*=0.71, *P*<0.0001), PINP (*r*=0.66, *P*<0.0001), TRACP5b (*r*=0.60, *P*<0.0001) and OPG (*r*=0.26, *P*<0.05) than CTX which demonstrated no significant association with VEGF and OPG, and only weak association with the other markers (*r*=0.26–0.78, *P*<0.05–0.0001). VEGF correlated weakly with MMP-2 and -7 and was highly associated with TIMP-1 (*P*<0.0001).

TGF*β* only weakly correlated with MMP-2, TIMP-1 and TRACP5b (*P*<0.01). OPG did not significantly correlated with the other markers, except slightly with MMP-9 (*r*=0.33, *P*<0.05) and TIMP-1 (*r*=0.45, *P*<0.001).

### Sensitivity and specificity of biochemical markers for detecting bone metastases

To investigate the diagnostic value of the biochemical markers to distinguish patients with breast cancer with and without bone metastases, ROC analyses were performed. As shown in Table 2, at the optimal cutoff value each marker had a similar diagnostic sensitivity with about 68–83% of patients correctly classified with or without bone metastases, except for TGF*β*1 and OPG, which demonstrated low sensitivity and specificity and a diagnostic value lower than 65% (Table 2). For most of the markers, the optimal cutoff which discriminates between patients with and without bone metastases is comparable to the upper limit of healthy controls as defined by the 95th percentile (Table 2).

When all markers were included in a multivariable discriminant analysis model, TRACP5b (*P*=0.009), CTX (*P*=0.03), VEGF (*P*=0.05) and ICTP (*P*=0.14) were significantly and independently associated with the presence of bone metastases. In this analysis, markers were included in the model when the associated *P*-value is <0.15 (see Materials and methods). This combination of markers allowed to correctly classifying 85% of patients with or without bone metastases.

### Biochemical markers and progression toward bone metastases

To investigate whether biochemical markers could predict which patients are at risk for developing bone metastases, patients with a primary breast cancer who did and did not progress toward bone metastases during follow-up have been compared.

As shown on Table 3, at the time of primary breast cancer, there was no statistical significant difference in any biochemical markers between patients who did and those who did not progress toward bone metastases. Serum TRACP5b levels were on average 95% higher in patients who will develop bone metastases compared to levels in patients who did not develop bone metastases although the difference did not reach statistical significance (*P*=0.08).

**Figure 5:**
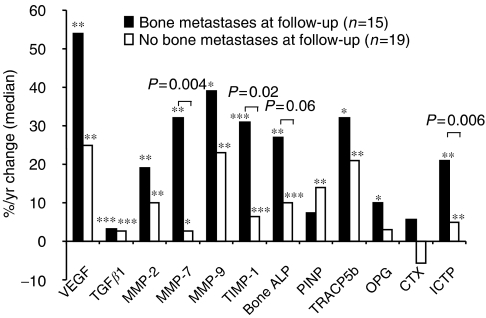
Changes of biochemical marker levels in patients with primary breast carcinoma who developed or remained free of bone metastases during a 3 years follow-up. The bars represent the changes of levels between the follow-up and the initial visits divided by the duration of follow-up and expressed as percentage of the initial value. The actual *P*-values indicated on the figure refers to the comparison of the percentage changes between the two groups. ^*^P<0.05, ^**^P<0.01, ^***^P<0.001 at follow-up *vs* baseline in each group separately.

**Figure 6:**
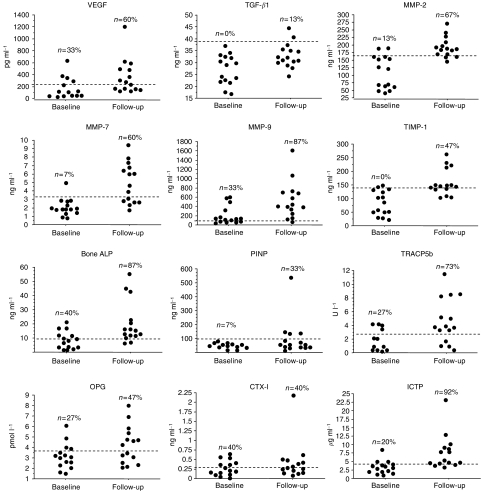
Individual values of biochemical markers of angiogenesis, tumour invasion and bone turnover in the 15 patients participating in the longitudinal evaluation who develop bone metastases during follow-up. For each marker, individual values are shown at the time of diagnosis of primary breast cancer (baseline) and at the time of second sampling before bone metastases was documented by bone scan (follow-up, see Patients and methods). Dotted lines indicate the upper limit of healthy controls (95th percentiles); the percentages represent the proportion of patients with values above the upper limit of healthy controls at baseline and at follow-up.

During the follow-up period, levels of all markers increased significantly in patients who developed bone metastases before they were documented by bone scan, except for PINP and CTX (Figure 5). The percentage of patients with values above the upper limit of controls was low at the time of diagnosis of primary breast cancer and increased in those patients who developed bone metastases for all markers except CTX-I (Figure 6). For patients who did not develop bone metastases all markers also increased significantly, except for OPG and CTX. The increase in the biochemical markers was higher in patients who progressed toward bone metastases when compared to those who did not, although the difference reached statistical significance only for MMP-7, TIMP-1 and ICTP and was borderline significant for bone ALP (Figure 5).

## DISCUSSION

This is one of the few studies which investigated a broad panel of biochemical markers reflecting the multiple biological processes involved in the formation of bone metastases associated with breast cancer. We investigated the association of these markers with bone metastases both in cross-sectional and longitudinal analysis. We found that biochemical markers of angiogenesis (VEGF), degradation of extracellular matrices (MMPs) and bone resorption were positively associated with bone metastases in breast carcinoma patients. Moreover, our data suggest that a few of these markers could be of clinical usefulness, since they have shown independent predictive value.

We found increased VEGF in breast cancer patient with bone metastases, but not in patients with a localised disease, which is consistent with previous studies (Adams *et al*, 2000; Coskun *et al*, 2003). Increased VEGF level was also associated with biochemical markers of osteoclastic activity and bone matrix degradation, which is in agreement with a recent study showing high that VEGF stimulates osteoclastic differentiation *in vitro* (Aldridge *et al*, 2005).

Our study investigated for the first time the serum levels of TGF*β*1 in patients with and without bone metastasis and identified that both breast cancer patients with and without bone metastases had significantly lower levels compared to healthy age-matched controls. Decreased TGF*β* levels in breast carcinoma patients is consistent with the inhibitory effect of endogenous TGF*β* on human breast cancer cells proliferation (Zugmaier *et al*, 1989; Arteaga *et al*, 1990). Other studies, however, have reported no significant difference of serum TGF*β*1 levels between breast cancer patients and healthy controls, indicating a need for further examination in larger studies (Sheen-Chen *et al*, 2001; Ivanovic *et al*, 2003; Lebrecht *et al*, 2004).

The role of MMPs in breast cancer initiation, invasion and metastasis is well established (Kleiner and Stetler-Stevenson, 1999; Duffy *et al*, 2000). MMP-9 has been found to be associated with poor prognosis and metastatic potential of breast carcinoma (Duffy *et al*, 1995). Similarly, high serum level of MMP-2 indicated adverse prognosis in node-positive breast carcinoma (Leppa *et al*, 2004). Our data confirm the involvement of MMP-2 and MMP-9 in promoting bone metastases. This is the first study showing a slight increase of circulating levels of MMP-7 in patients with breast cancer and bone metastases. Interestingly, a recent immunohistochemistry study has reported expression of MMP-7 in about 50% of human breast cancer cells which correlates with tumour invasion (Mylona *et al*, 2005).

Osteoprotegerin, a pivotal regulator of the osteoclastic activity, (Hofbauer and Schoppet, 2004) has been found to be increased in prostate cancer patients with bone metastases – but not in those with localised diseases (Brown *et al*, 2001; Jung *et al*, 2004). In our study, breast cancer patients with or without bone metastases had increased OPG levels compared to healthy controls, contrasting with the absence of elevation found by Lipton *et al* (2002). The reasons for these discrepancies are unclear, but they could be attributed to patient population characteristics and/or specificity of assays for the various circulating forms of OPG (Yano *et al*, 1999).

To assess bone formation and bone resorption, we measured the currently available most sensitive and specific biochemical markers (Garnero and Delmas, 2003). For formation, we assessed serum bone ALP, a specific enzyme of the osteoblastic cells and serum PINP, which reflects the synthesis of the main bone matrix protein. For bone resorption, we evaluated serum TRAPC5b a specific enzyme of the osteoclastic cells, and serum CTX and ICTP, two fragments of type I collagen degradation. Previous studies have evaluated only a few of these markers (Fohr *et al*, 2003), but our study is one of the first which measured concomitantly all of them in the same patients. We found that ICTP and CTX were only modestly associated and ICTP – but not CTX – was highly correlated with MMPs, in particular, MMP-2. These findings suggest that in patients with metastatic bone disease, serum CTX and ICTP could reflect distinct biological pathways of bone resorption, consistent with *in vitro* studies showing that ICTP – but not CTX – is directly released from bone collagen matrix by MMPs, while CTX could be released from bone collagen by other proteolytic enzymes, such as cathepsin K (Garnero *et al*, 2003b).

To assess the potential clinical usefulness of biochemical markers, we have determined their sensitivity, specificity and accuracy to distinguish patients with and without bone metastases. At the optimal cutoff value determined from ROC analyses, we found that each marker has a similar diagnostic accuracy except for TGF*β*1 and OPG, which demonstrated very low sensitivity. It is difficult to compare the cutoff values we determined in this study with previous reports because these are highly dependent on the actual assay used which can differ between studies for a same marker and which are not standardised. Interestingly, however, the optimal cutoff values determined by ROC analyses were very close to the upper limit of healthy controls for most markers. Thus, if confirmed in other larger studies, using the 95th percentile of healthy age-matched could represent an adequate cutoff to best differentiate patients with breast cancer with or without bone metastases. In multivariable analyses, we found that besides markers of bone resorption, which are specific to bone, only VEGF was independently associated with bone metastases, probably because it mediates biological processes involved at multiple steps of bone metastasis formation.

Very few studies have investigated whether biochemical markers measured in patient with primary breast cancer could predict the development of bone metastasis. In a study of 388 patients with localised breast cancer, Diel *et al* (1999) reported that breast carcinoma patients with increased level of bone sialoprotein (BSP) were at a higher risk of devolving bone metastases over the following 20 months. More recently, in a small case–control study, Seibel *et al* (2002) found no difference in several biochemical markers of bone turnover including bone ALP, osteocalcin, PICP, serum CTX and the urinary excretion of deoxypyridinoline between the 11 patients who developed bone metastases and the 44 controls. That study, however did not investigate serum TRACP5b and ICTP. Among the markers we evaluated in our study, only TRACP5b was increased in patients with a primary carcinoma who progressed toward bone metastases, when compared to patients who did not develop bone metastasis. However, the difference did not reach statistical significance, probably because of the limited number of subject we evaluated. Interestingly, during follow-up serum MMP-7, TIMP-1 and ICTP increased even before bone metastases could be documented by bone scans. If confirmed in larger prospective studies, these findings suggest that biochemical abnormalities may be detected by serum tests before bone metastases are being documented by bone scintigraphy.

In conclusion, this cross-sectional and longitudinal evaluation of multiple circulating biochemical markers reflecting the different biological processes involved in bone metastases development indicate that markers of bone resorption were the most sensitive to detect the presence of bone metastasis in breast cancer patients. If confirmed in larger prospective studies, regular monitoring of patients with primary breast cancer by an optimal combination of circulating biochemical markers may allow detecting individuals with bone metastases at an early stage, potentially before detection with bone scintigraphy.

## Figures and Tables

**Table 1:** Characteristics of women with breast cancer and without bone metastases

**Characteristics**	**Patients with primary breast cancer *n*=29**	**Patients with breast cancer and bone metastases *n*=28**
Age (mean, s.d.); year	53, 10	55, 11
Weight (mean, s.d.), kg	65.4, 11.3	69.4, 11.7
Height (mean, s.d.), cm	164, 5.6	160, 10.9
		
*Tumour size (%)*		
T1	69	50
T2	28	29
T3	0	7
T4	0	11
Unknown	3	3
		
*Stage (%)*		
I	93	11
II	7	32
III	0	3
IV	0	54
		
*Histology (%)*		
Ductal carcinoma	58	64
Lobular carcinoma	7	7
Other	14	7
Unknown	21	22
		
*Axillary nodal status (%)*		
N–	97	64
N+	3	36
Oestrogen receptor positivity (%)	52	82
		
*Therapy (%)*		
Radiation	16	0
Chemotherapy	21	22
Hormonal	0	22
Unknown	63	56

**Table 2:** Diagnostic value of biochemical markers to distinguish breast carcinoma patients with bone metastases from those without bone involvement

**Marker**	**Cutoff value**	**AUC**	**Sensitivity (%)**	**Specificity (%)**	**Diagnostic accuracy (%)**	**95th Percentile of healthy controls**
VEGF (pg/ml)	478	0.768	43	100	78	202
TG*β*1 (ng/ml)	41	0.511	18	95	65	39.8
MMP-2 (ng/ml)	169	0.900	83	85	85	174
MMP-7 (ng/ml)	4.62	0.752	43	98	76	3.41
MMP-9 (ng/ml)	376	0.702	79	66	71	144
TIMP-1 (ng/ml)	176	0.768	46	91	74	149
Bone ALP (ng/ml)	11.5	0.858	79	82	81	9.6
PINP (ng/ml)	80	0.815	46	98	78	79
TRACP 5b (U/l)	3.05	0.856	64	95	83	2.87
OPG (pmol/l)	5.33	0.600	32	84	64	3.67
CTX (ng/ml)	0.382	0.814	95	75	68	0.353
ICTP (*μ*g/l)	4.47	0.903	86	82	83	4.25

Receiving operating curves (ROC) were generated and the area under the curve (AUC) sensitivity, specificity were determining at the cutoff providing the highest diagnostic accuracy for each individual marker.

**Table 3:** Levels of biochemical markers at the time of diagnosis of primary breast carcinoma in patients who developed and in those who remained free of bone metastases during the following 3 years

	**Bone metastases at baseline**		
**Marker**	**Yes (*n*=15)**	**No (*n*=19)**	***P*-value**
VEGF (pg/ml)	114 (242)	160 (154)	0.61
TGF*β*1 (ng/ml)	29.1 (10.2)	30.5 (8.0)	0.16
MMP-2 (ng/ml)	121 (97)	116 (63	0.39
MMP-7 (ng/ml)	1.8 (1.1)	2.5 (1.1)	0.06
MMP-9 (ng/ml)	127 (210)	226 (188)	0.14
TIMP-1 (ng/ml)	87 (80)	106 (58.9)	0.11
Bone ALP (ng/ml)	8.2 (8.5)	8.3 (6.6)	0.60
PINP (ng/ml)	47 (24)	30 (27)	0.20
TRACP5b (U/l)	3.7 (2.1)	1.9 (1.3)	0.08
OPG (pmol/l)	3.1 (1.4)	3.0 (2.5)	0.91
CTX (ng/ml)	0.2 (0.3)	0.2 (0.1)	0.57
ICTP (*μ*g/l)	3.1 (2.2)	3.2 (1.3)	0.78

Data are expressed as median (interquartile).
